# Pharmacokinetic/Pharmacodynamic Profiles of Tiamulin in an Experimental Intratracheal Infection Model of *Mycoplasma gallisepticum*

**DOI:** 10.3389/fvets.2016.00075

**Published:** 2016-09-06

**Authors:** Xia Xiao, Jian Sun, Tao Yang, Xi Fang, Jie Cheng, Yan Q. Xiong, Ya-Hong Liu

**Affiliations:** ^1^National Reference Laboratory of Veterinary Drug Residues (SCAU), College of Veterinary Medicine, South China Agricultural University, Guangzhou, China; ^2^Jiangsu Co-Innovation Centre for Prevention and Control of Important Animal Infectious Diseases and Zoonoses, Yangzhou, Jiangsu, China; ^3^Los Angeles Biomedical Research Institute, Harbor-UCLA Medical Center, Torrance, CA, USA; ^4^David Geffen School of Medicine, UCLA, Los Angeles, CA, USA

**Keywords:** tiamulin, *M. gallisepticum*, *in vivo* PK/PD, chicken

## Abstract

*Mycoplasma gallisepticum* is the most important pathogen in poultry among four pathogenic *Mycoplasma* species. Tiamulin is a pleuromutilin antibiotic that shows a great activity against *M. gallisepticum* and has been approved for use in veterinary medicine particularly for poultry. However, the pharmacokinetic/pharmacodynamics (PK/PD) profiles of tiamulin against *M. gallisepticum* are not well understood. Therefore, in the current studies, we investigated the *in vivo* PK/PD profiles of tiamulin using a well-established experimental intratracheal infection model of *M. gallisepticum*. The efficacy of tiamulin against *M. gallisepticum* was studied in 8-day-old chickens after intramuscular (i.m.) administration at 10 doses between 0–80 mg/kg. Liquid chromatography-tandem mass spectrometry (LC-MS/MS) was used to evaluate the PK parameters of tiamulin following i.m. administration at doses of 5, 40, and 80 mg/kg in *Mycoplasma gallisepticum*-infected neutropenic chickens. Real-time PCR (RT-PCR) was used for quantitative detection of *M. gallisepticum*. The MIC of tiamulin against *M. gallisepticum* strain S6 was 0.03 μg/mL. The PK/PD index, AUC_24h_/MIC, correlated well with the *in vivo* antibacterial efficacy. The *in vivo* data suggest that animal dosage regimens should supply AUC_24h_/MIC of tiamulin of 382.68 h for 2 log_10_ ccu equivalents *M. gallisepticum* reduction. To attain that goal, the administered dose is expected to be 45 mg/kg b.w. for treatment of *M. gallisepticum* infection with an MIC_90_ of 0.03 μg/mL.

## Introduction

*Mycoplasma gallisepticum* (*M. gallisepticum*), a multi-host pathogen, is the most pathogenic agent of chronic respiratory disease (CRD) in poultry and turkey (1, 2). It can also be transmitted from poultry to house finches as well as other similar species and cause outbreaks of upper respiratory disease (3). Because it produces vast losses in the poultry industry, *M. gallisepticum* is considered as the most economically important pathogen of the four pathogenic *Mycoplasma* species (4).

Current means of controlling *M. gallisepticum* infections among avian species include biosurveillance practices, vaccines, and medication (5). Despite control measures, *M. gallisepticum* may be present in chick flocks that are maintained for long growing periods with minimal biosecurity. Some live attenuated *M. gallisepticum* vaccines are approved for use only within the commercial egg layer industry (6). The effectiveness of other subunit vaccines may be limited due to efficacy and associated costs (7). Although some vaccines are effective, *M. gallisepticum* outbreaks within vaccinated flocks has been observed (7), suggesting better medication control are required.

Antibiotics have been extensively used in the areas with intensive and varied population of poultry flocks for controlling *M. gallisepticum* or other pathogen infections (8). The use of antimicrobial agents has been considered as an economic method for controlling *M. gallisepticum* infections (8). Several kinds of antibiotics (macrolides, tetracyclines, fluoroquinolones, and pleuromutilin) have displayed good activity against *M. gallisepticum* (9, 10). However, it has also been noticed that antibacterial usage over time can induce resistance in this organism (11, 12).

Tiamulin is a semisynthetic derivative of the diterpene antibiotic pleuromutilin used in swine and poultry for treatment and prophylaxis of dysentery, pneumonia, and mycoplasmal infections (13). Although it has been used over 30 years, no significant changes of susceptibility on this pathogen were observed previously (11, 14). However, one recent investigation indicated that tiamulin resistant isolates were seen after tiamulin treatment (15). Therefore, it is important to study the optimal tiamulin regimen in order to maximize antibacterial activity and to prevent emergence of resistance. It is well-known that pharmacokinetic (PK) and pharmacodynamics (PDs) profiles of antibiotics provide useful information in the establishment of optimal dose regimens for better clinical setting management and prohibit resistance emergence (16, 17). To our best knowledge, PK/PD profiles of tiamulin against *M. gallisepticum* are very limited. Therefore, in the current experiment, we performed *in vivo* PK/PD studies of tiamulin against *M. gallisepticum* after intramuscular (i.m.) administration. Though the i.m. administration has not been approved for tiamulin in chicks, this study would provide foundation for tiamulin injectable formulation for chicks in future. The goals of the present investigations were to: (1) evaluate the PK profiles and dose proportionality of tiamulin in an intratracheal infection animal model; (2) provide the magnitude of the PK/PD index AUC_24h_/MIC for different extent of efficacies; and (3) establish a rational dosage regimen that optimizes tiamulin efficacy with respect to bacteriological and clinical outcomes.

## Materials and Methods

### Bacteria, Chemicals, Susceptibility Assay, and Animals

A well-characterized *M. gallisepticum* standard virulent strain S6 was purchased from the Chinese Veterinary Microorganism Culture Collection Center (Beijing, China). Tiamulin Fumarate (>99%) was kindly supplied by the Hebei Yuanzheng Pharmaceutical Company (Hebei, China). The MIC of tiamulin on the strain S6 was determined by a standard micro-dilution method according to recommended protocols (18, 19). Three hundred fifty Sanhuang chickens of 1-day-old weighting 35~45 g supplied by Guangdong Academy of Agricultural Sciences (Guangzhou, Guangdong, China) were used in this experiment. Birds were free of *M. gallisepticum* and fed with clean water and antibacterial-free fodder.

### *In Vitro* Standard DNA Preparing

An *in vitro* DNA standard curve was established according to our previous report (19). Briefly, 36 h incubated *M. gallisepticum* medium was centrifuged for 10 min at 1,500 rpm and then resuspending the pellet in 0.6 mL fresh *M. gallisepticum* medium. 0.1 mL of the sample was serial diluted for bacteria counting by culture method [color change unit per millimeter (ccu/mL)]. Meanwhile, DNA was isolated from the sample and serial 10-fold dilutions (10^0^–10^−6^) prepared from the 0.6 mL sample with a bacteria DNA kit (Omega Bio-tek, Inc., Norcross, GA, USA). The DNA copies of *M. gallisepticum* were determined by real-time PCR (RT-PCR) (20). The DNA standard curve was plotted by the number of *M. gallisepticum* calculated from the culture method and cycle threshold (*C*_t_) values obtained using RT-PCR results.

### Neutropenia Model

Two days post arrival, the chicken neutropenia model published by our laboratory (19) was established in this study in order to eliminate the immunity variance of different chickens and study the efficacy of tiamulin solely, *via* intramuscular administration of cyclophosphamide at 60 mg/kg for 3 days (21). Birds were severely granulocytopenic (absolute leukocyte count <1,000/mm^3^) and remained so for 8 days after the third injection of cyclophosphamide. The South China Agriculture University Animal ethics committee approved all *in vivo* experiments with an approved number of 2014-08. In addition, all husbandry practices and experimental operations were performed with full consideration of animal welfare.

### *M. gallisepticum* Intratracheal Infection Model

*M. gallisepticum* mainly invades the respiratory system in chickens. Thus, an *M. gallisepticum* intratracheal infection model was utilized in this study according to Xia’s report (19). Briefly, 24 h post the last dose of cyclophosphamide, 0.2 mL of solution containing approximately 10^8^ color change unit (ccu) of the *M. gallisepticum* strain was inoculated intratracheally to neutropenic chickens for 3 days [95% infective dose (ID_95_) for the studied strain]. Initial pathogen loading was quantified according to our established method described in our previous report (19). Briefly, at 24 h after the last infection dose, chickens were euthanized and trachea, air sac, and lungs were collected, homogenized in 2 mL PBS and centrifuged at 500 rpm for 5 min. An aliquot of 0.5 mL supernatant was used for DNA extraction with a bacterial DNA kit (Omega Bio-tek, Inc., Norcross, GA, USA) as described above. DNA copies of *M. gallisepticum* in these samples were measured by RT-PCR as described above. The amount of *M. gallisepticum* was calculated using the DNA copies *via* the *in vitro* standard curve.

### Determination of DNA Copies of *M. gallisepticum* Using RT-PCR

The method determinating DNA copies of *M. gallisepticum* in different samples by RT-PCR were identical with our previous report (19). All RT-PCR reactions were performed on a BIO-RAD iQ 5 (Bio-Rad Laboratories, Inc., USA) using the SYBR premix Ex Taq™ (TaKaRa, Shiga, Japan). *C_t_* values were defined as the cycle number yielding a maximum value of the second derivative of the amplification curve of the sample. Samples were defined as positive when both a measurable *C_t_* and the expected *T_m_* (±0.5°C) were seen. The standard samples and a negative control (elution buffer) were included in each run.

### Tiamulin Pharmacokinetics in Neutropenic Intratracheal Infection Model

The infected neutropenic chickens were administrated with tiamulin intramuscularly at single doses of 5, 40, or 80 mg/kg b.w. 1.5 mL blood was sampled from the neck vein at 5, 10, 30 min, 1, 2, 4, 6, 8, 12, and 24 h after drug administration (10 chickens/time point). Blood samples were incubated immediately at room temperature for 1 h and then placed in 4°C for 2 h to enable clot retraction. Serum was obtained by centrifuging at 3,000 rpm for 10 min and frozen at −20°C immediately until analysis within 2 weeks. Tiamulin was extracted from serum by acetonitrile with a proportion of 2:1. Concentrations of tiamulin in serum were determined *via* a high-performance liquid chromatography-tandem mass spectrometry (LC-MS/MS) method, which was developed by our group and reported previously (22, 23). The recovery and precision were calculated by analysis of spiked samples at three concentration levels (5 replicates of each concentration). Mean recoveries of tiamulin that spiked at three concentration levels were in the range of 86.0–92.7%. The limit of quantitation (24) was confirmed at 2.5 ng/mL. The coefficient of correlation (*r*^2^) was 0.9995 for the linear range of 2.5–500 ng/mL. The intra-day and inter-day coefficients of variation were determined to be 5.6 and 10.7%.

### Efficacy of Tiamulin in Neutropenic Chicken Intratracheal Infection Model

To evaluate the efficacy of tiamulin at 24 h post-three day infections, either 0.85% NaCl (controls) or tiamulin at 5, 10, 20, 30, 40, 50, 60, 70, or 80 mg/kg were administrated to infected neutropenic chickens intramuscularly once daily for 3 days (five chickens/dose). At 24 h after the last drug administration, the amounts of *M. gallisepticum* in each chicken were calculated using the method described above.

### Pharmacokinetics and Pharmacodynamics Analysis

The PK profiles of tiamulin were analyzed by the non-compartmental model with uniform weighting using the WinNonlin software (version 6.1; Pharsight, CA, USA). The surrogate marker of antibacterial activity, AUC_24h_/MIC were calculated using *in vitro* MIC value and PK parameters obtained from three doses of i.m. administrations of tiamulin. The bacteria loading for each animal was calculated according to *C_t_* values and the *in vitro* standard DNA curve. The efficacy of tiamulin was evaluated by the reduction of *M. gallisepticum* compared to the initial bacteria count before drug treatment. The *in vivo* PK/PD relationship of tiamulin against *M. gallisepticum* was studied using the sigmoid *E*_max_ model WINNONLIN software (version 6.1; Pharsight, CA, USA) with the equation as follows:

$$E=E_{0}+\frac{E_{\text{max}}\times C_{e}^{N}}{{\text{EC}}_{50}^{N}+C_{e}^{N}}$$

where *E*_0_ is the change in log_10_ ccu equivalents/mL in the control sample (absence of tiamulin), *E*_max_ is the difference in log_10_ ccu equivalents/mL of the greatest amount of kill, *C_e_* is the AUC_24h_/MIC in the effect compartment, EC_50_ is the AUC_24h_/MIC value producing a 50% reduction in bacterial counts, and *N* is the Hill coefficient that describes the steepness of the curve (25).

### Dosage Calculation

In order to deduce a more rational regimen, the general formula was employed to estimate dosages for different magnitudes of efficiency (26).

$$\text{Dose}=\frac{{\text{CL}}_{\text{per hour}}\times ({\text{AUC}}_{24}/\text{MIC})\times {\text{MIC}}_{90}}{F\times f_{u}}$$

where dose is the optimal dose (milligram/kilogram/day), CL is the body clearance (liter/kilogram/day), AUC/MIC is the breakpoint marker for the desired effect (hour), MIC_90_ is the MIC inhibiting 90% of strains (milligram/liter), *F* is the bioavailability, and *f_u_* is the free drug fraction.

## Results

### Susceptibility Testing

The MIC of tiamulin against the studied strain was 0.03 μg/mL.

### *In Vitro* Standard DNA Curve

**Figure 1:**
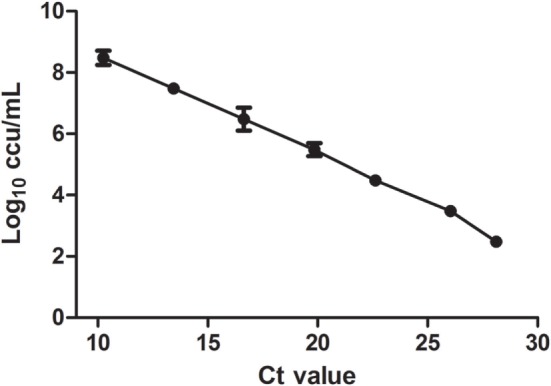
***In vitro* DNA standard curve**. Relationship of DNA standards between *C_t_* value and culture results (Log_10_ ccu/mL). Mean of three different RT-PCR runs.

The correlation between the *C*_t_ values and log_10_ ccu/mL reached statistic significance with the equation of *y* = −0.3087*x* + 10.44 and *R*^2^ of 0.9988 (Figure 1). The limitation of detection was 3 × 10^2^ equivalents ccu/mL. The recovery rates at different dilutions were 56.5 ± 5.1%.

### *M. gallisepticum* Intratracheal Infection Model

Major clinical symptoms, including depression, mouth breathing, eye closures, were observed from the infected animals. In addition, histopathology studies showed that airsacculitis as the cardinal symptom of *M. gallisepticum* infection was observed in 95% infected chickens. The mean *M. gallisepticum* load was 2.2 × 10^6^ ccu equivalents/mL for all inoculated chickens. The morbidity and mortality rates were 95 and 17% at 5 days post-infection, respectively. Neither clinical symptoms nor airsacculitis were observed in the control animals. Bacteriological assays were also negative in the control group.

### Tiamulin Pharmacokinetics Profiles in Neutropenic Intratracheal Infection Model

**Figure 2:**
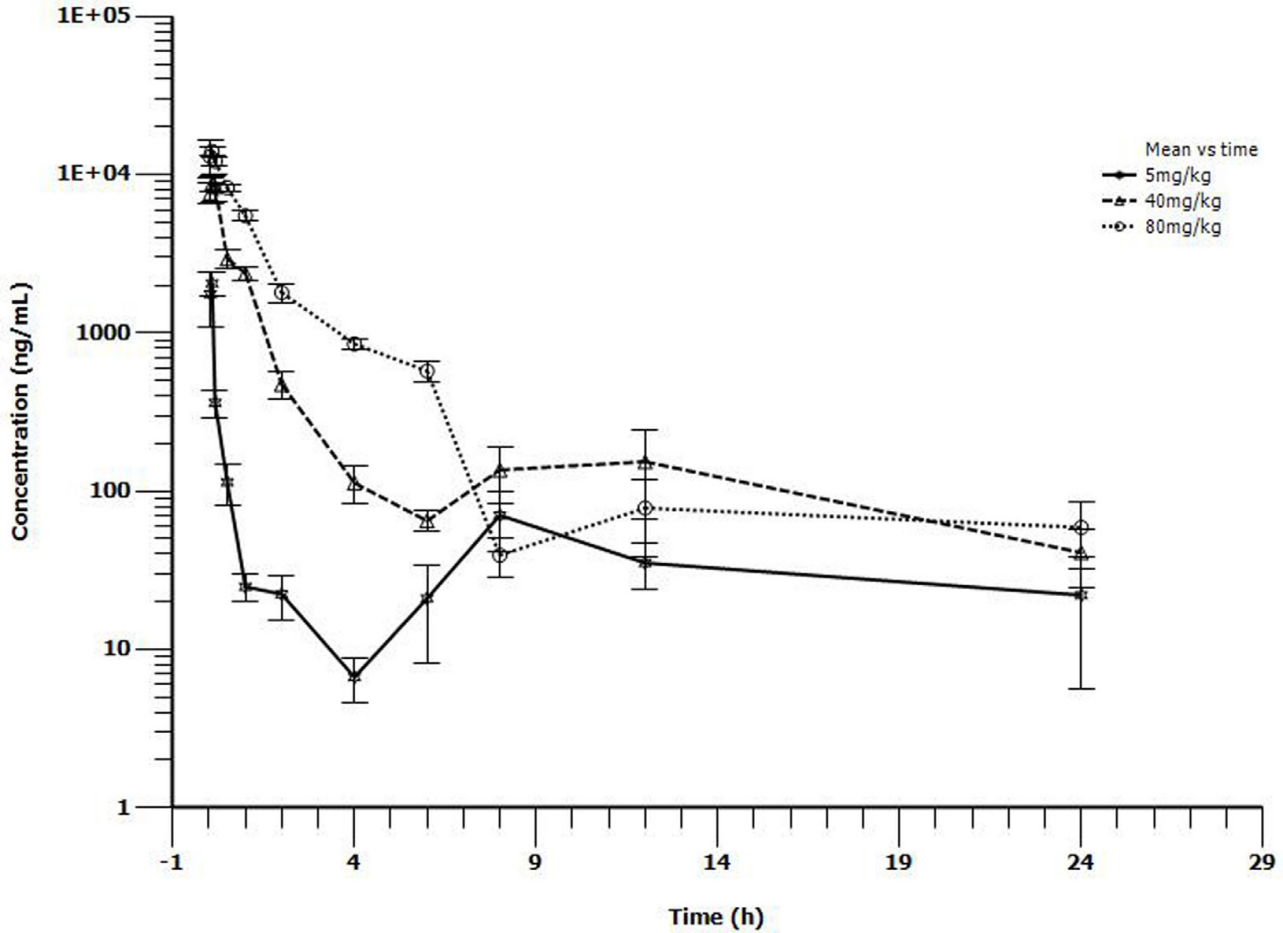
**Serum concentrations of tiamulin following i.m. administration at a single dose of 5, 40, or 80 mg/kg in *M. gallisepticum* intratracheal infection model (*n* = 10/time point)**.

**Figure 3:**
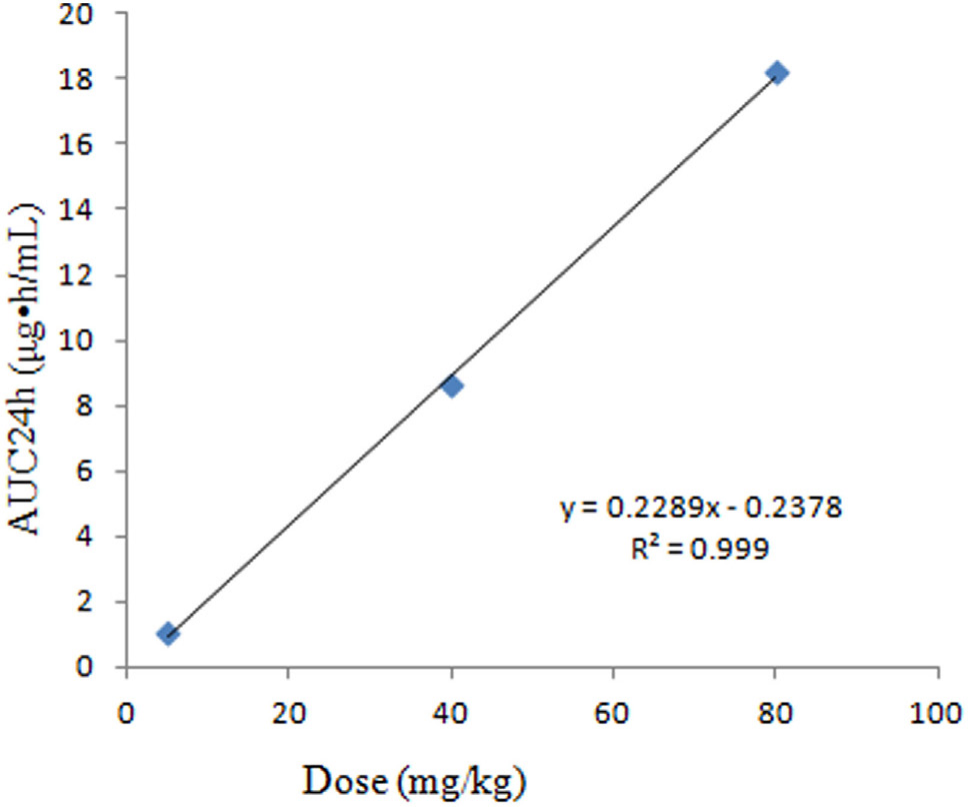
**Linear regression plots between administered dose and AUC_24h_ values**.

The main PK parameters are presented in Table 1 and Figure 2. The *C*_max_ were 2.05, 8.8, and 14 μg/mL for 5, 40, and 80 mg/kg doses, respectively, which were observed at 0.167 h after administration. The half-life (*T*_1/2β_) was about 1.24 h for all three different doses. A second peak was observed for all the doses administered at 8–12 h. Importantly, a significant correlation between doses and AUC_24h_ was observed (*R*^2^ = 0.999, Figure 3). As the AUC_24h_ were increasing in a dose-dependent manner from 5 to 80 mg/kg, the AUC_24h_ of other dose regimens were calculated according to the linear relationship. The parameters of CL were parameterized as CL/F because of the extravascular administration while MRT_last_ was the ratio of AUMC_last_/AUC_last_.

**Table 1:** **Pharmacokinetic parameters of tiamulin in serum following intramuscular administration at a single dose of 5, 40, or 80 mg/kg in *M. gallisepticum*-infected chickens (*n* = 10/group)**.

Parameters (units)	5 mg/kg	40 mg/kg	80 mg/kg
*C*_max_(μg/mL)	2.05	8.8	14
*T*_max_(h)	0.167	0.167	0.167
*T*_1/2β_ (h)	1.15	1.39	1.17
AUC_24h_ (μg h/mL)	1.07	8.61	18.21
CL/F (L/h/kg)	4.53	4.6	4.37
MRT_last_ (h)	8.15	3.46	2.33

*C_max_, maximum serum concentration; T_max_, time of maximum serum concentration; T_1/2β_, elimination half-life; AUC_24h_, 24 h area under serum concentration-time curve; CL/F, clearance divided by bioavailability; MRT_last_, residence time*.

### *In Vivo* Efficacy of Tiamulin in Neutropenic Chicken Intratracheal Infection Model

**Figure 4:**
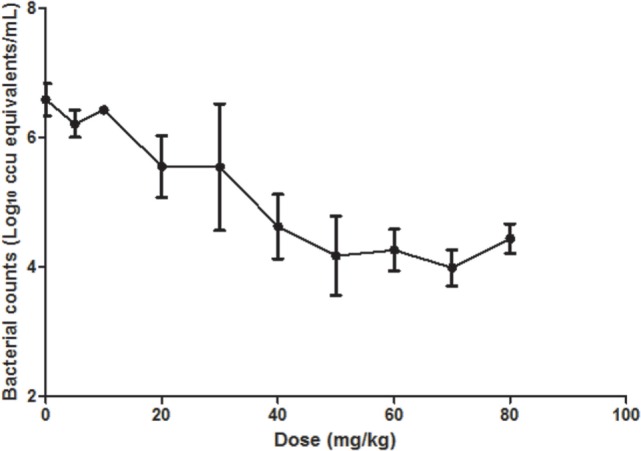
**The calculated *in vivo M. gallisepticum* counts after tiamulin treatment**. (*n* = 4–7/dose).

The *C_t_* values of respiratory tissues of chickens administrated with doses from 5 to 80 mg/kg increased with the increasing of doses, implying that the bacteria loading decreased with the dose increasing. The *C_t_* value increased sharply between dosages of 10 and 50 mg/kg while gently from 50 to 80 mg/kg (Figure 4). Limbs Twitch was observed in chickens treated with doses of 60, 70, 80 mg/kg for about 10 min; however, no death emerged.

### Tiamulin PK/PD Profiles

**Figure 5:**
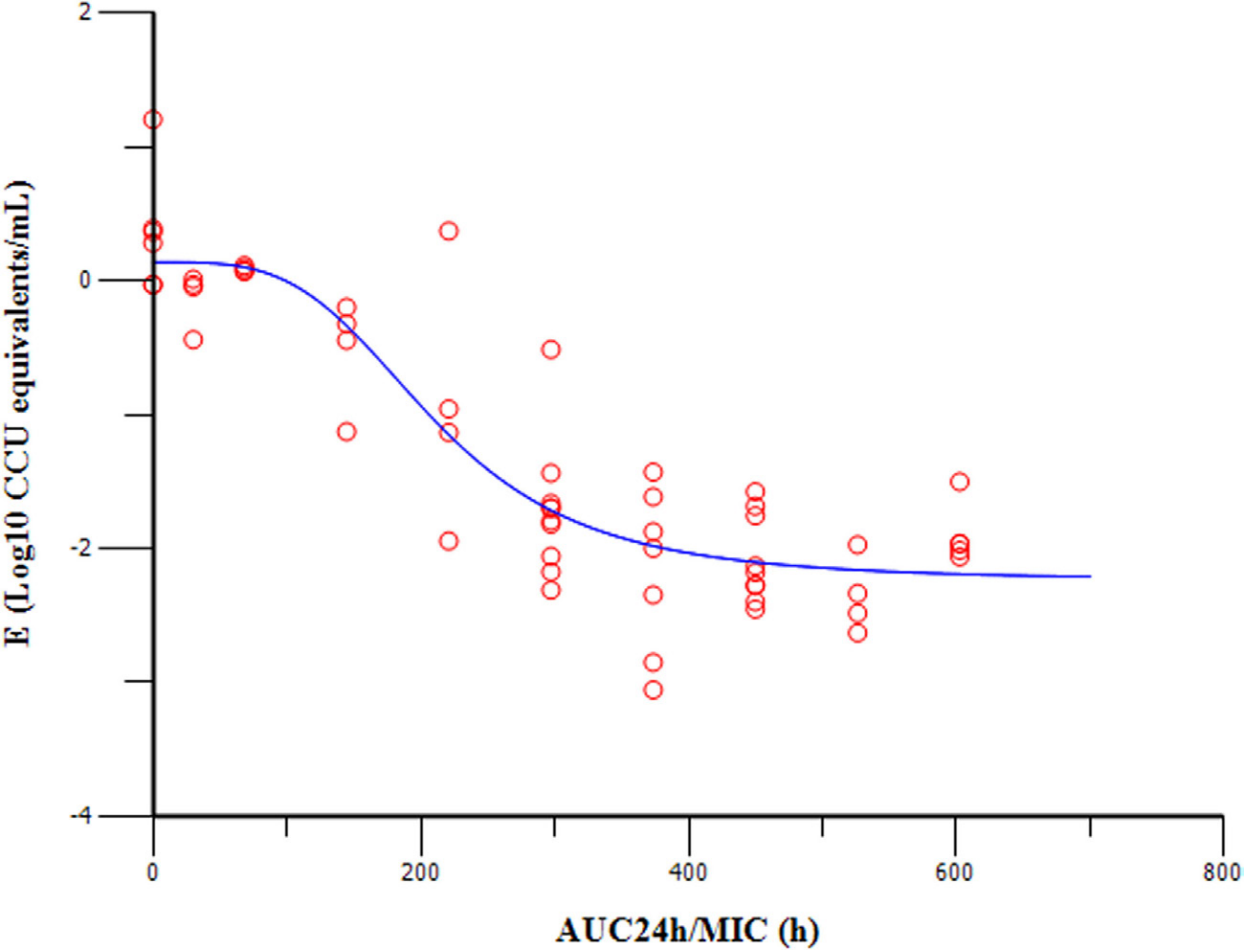
**Sigmoid *E*_max_ relationships of tiamulin between antimycoplasmal effect (*E*, log_10_ ccu equivalents/mL) and *in vivo* AUC_24h_/MIC ratio against MG in serum of chickens**.

The PK/PD indices AUC_24h_/MIC was integrated using the PK parameters, dose proportionality, and MIC data. The effect (*E*) was calculated as the reduction of *M. gallisepticum* using the unit of Log_10_ ccu equivalent/mL. The relationship between the ratio of AUC_24h_/MIC and antibiotic efficacy was described using the Sigmoid *E_max_* model (Figure 5). The AUC_24h_/MIC corrected well with the effects (*R*^2^ = 0.8979). The values of AUC_24h_/MIC for mycoplasmastasis (0 log_10_ ccu/mL reduction), for 1 log_10_ ccu reduction and 2 log_10_ ccu reduction are 98.98, 206.56, 382.58, respectively (Table 2). The EC_50_ was 211.19 h, and the slope of the graph (*N*) was 3.68.

**Table 2:** **Tiamulin PK/PD analysis with the parameter of AUC_24h_/MIC in *M. gallisepticum* intratracheal infection model**.

Parameter	Value	SD
*E*_max_ (Log_10_ ccu equivalents/mL)	2.38	0.24
*E*_0_ (Log_10_ ccu equivalents/mL)	0.14	0.13
EC_50_ (h)	211.19	20.3
AUC_24h_/MIC for 0 log_10_ ccu equivalents/mL reduction (h)	98.98	
AUC_24h_/MIC for 1 log_10_ ccu equivalents/mL reduction (h)	206.56	
AUC_24h_/MIC for 2 log_10_ ccu equivalents/mL reduction (h)	382.58	
Slope (*N*)	3.68	1.2

*E_0_ is the change in Log_10_ ccu equivalents/mL after 24 h incubation in the control sample (absence of drug) compared with the initial inoculum. E_max_ is the difference in effect of the greatest amount of kill. EC_50_ is the AUC_24h_/MIC value producing a 50% reduction in bacterial counts from the initial inoculum. AUC_24h_/MIC is the 24 h area under concentration-time curve/minimum inhibitory concentration ratios. N is the Hill coefficient that describes the steepness of the AUC_24h_/MIC-effect curve*.

### Dosage Calculation

To calculate dosages, the bioavailability was taken into account owing to the extravascular route of administration, and the free drug fraction was not required for using PD data generated *in vivo*. In order to provide a dosage regimen attaining maximum effect for chickens infected with *M. gallisepticum* with an MIC_90_ of 0.03 μg/mL, a dose of 45 mg/kg for 3 days was recommended.

## Discussion

*M. gallisepticum*, a highly transmissible, persistent pathogen in chickens, turkeys, and some wild birds, causes considerable economical losses to the poultry industry all over the world (27). Burch and Valks (28) had clarified that tiamulin possessed excellent activity against 241 *M. gallisepticum* strains by comparing the MIC of tiamulin against *M. gallisepticum* and the *C*_max_ or concentration at steady state (*C*_ss_) in chicken blood in 2002 (28). However, its chemotherapeutic properties are not well described. In addition, the use of PK/PD model to identify the PD activity by integrating the PK characters, MIC, and pathogen loading outcome has proven helpful in designing rational dosage regimens in humans and animals (29, 30). Burch and Alvarez (31) evaluated the relationship between *C*_max_/MIC or AUC/MIC and prevention or treatment results of tiamulin in *M. gallisepticum* infection in 3-week-old chickens (31). The PK/PD parameters in his study were with some rough as he used the reported PKs of tiamulin in 7-week-old health chickens to predict that in 3-week-old *M. gallisepticum*-infected chickens. And the PDs of that study was evaluated by scores of lesions, and whether *M. gallisepticum* was isolated or not from air sacs. It is known that isolation of *M. gallisepticum* is easy to influence by overgrowth of faster growing *Mycoplasma* species or impeded by other organisms or no growth in subculture, so it may not be a good method to judge the PDs. What’s more, the study did not calculate the PDs from the aspect of bacteria loading reduction, which is important in PK/PD study. Thus, in this study, *in vivo* PK/PD profiles of tiamulin were established using PK characters in infected chickens, MIC, and pathogen-loading outcome.

This was the first report about the PKs of tiamulin in *M. gallisepticum* intratracheal-infected chicks. Tiamulin was rapidly absorbed with the peak concentrations for 5, 40, 80 mg/kg dose of 2.05, 8.8, 14.0 μg/mL achieved at 0.167 h. The half-life was in the range of 1.15–1.39 h, which was lower than the value reported in dogs (4.7 h) (32), which indicated that the elimination of tiamulin was of significant difference among species. The rate and extent of absorption was constant among the tested doses with the values of clearance divided by bioavailability (CL/F) of 4.53, 4.6, 4.37 L/h/kg, respectively. That is to say, tiamulin showed dose-dependent PKs when given as a single i.m. dose of 5, 45, or 80 mg/kg b.w. This was in accordance with the report that tiamulin showed dose proportionality in the range of 10–25 mg/kg b.w. in dogs (32). This phenomenon was also confirmed in our previously research with valnemulin, which is also a semisynthetic derivative of the diterpene antibiotic pleuromutilin (19). A second peak was observed for all the doses administration at 8–12 h. Tiamulin mainly accumulates and metabolizes *via* liver. It was speculated that, according to the chemical structure, a relatively high liposolubility of tiamulin would result in enterohepatic recycling, which is possibly the main reason that the second peak was observed (33).

The parameters of tiamulin (AUC_24h_) that showed dose proportionality in the range of 5–80 mg/kg following i.m. administration allowed us to calculate the AUC_24h_ for other dosage administrations. As reported for other pleuromutilin derivatives (34, 35), the PK/PD surrogate of pleuromutilin derivatives is AUC_24h_/MIC. The data from the present multiple dosage studies of tiamulin confirmed the conclusion that the AUC_24h_/MIC was the PK/PD index of tiamulin. The values of AUC_24h_/MIC for mycoplasmastasis (0 log_10_ ccu equivalents reduction), activity of 1 log_10_ ccu equivalents reduction, and 2 log_10_ ccu equivalents reduction were 124, 205, and 327 h, respectively, which were much lower than those obtained from an *in vivo* study of valnemulin (28,820, 38,030, and 56,256 h, respectively) (36). One possible reason for the differences was that the concentration in serum was not that real one act on *M. gallisepticum* as the infection site is air sac or respiratory system, and antibiotic concentrations in air sac or lung are usually different from those in serum (14). Another reason could be that the distribution volume of timulin was much wider than that of valnemulin in chickens, which means that the tissue concentration of tiamulin maybe higher than that of valnemulin (36). Tiamulin did not reach a mycoplasmacidal effect (3 log_10_ ccu equivalents reduction), which was in accordance with Burch and Alvarez’s (31) results that even tiamulin concentration far exceeded the MBC could not eliminate the pathogen in treatment procedure (31).

As was observed in this investigation, doses over 60 mg/kg would trigger neuro-toxicity. The calculated dose of 45 mg/kg for 3 days was sufficient for clinical treatment of *M. gallisepticum* infection with an MIC_90_ of 0.03 μg/mL. This regimen was in the range of recommended doses for drinking water (30–60 mg/kg) in poultry but more than twice of the recommended dose range for i.m. administration (10–20 mg/kg) in pigs (13). It was also lower than the neuro-toxicity dose. Though the i.m. administration has not been approved for tiamulin in chicks, this result would provide foundation for tiamulin injectable formulation for chicks in future.

The conventional method for evaluating the efficacy of an antibacterial agent is bacteria counting by culture method. However, the cultivation technique is expensive and laborious and time consuming (37). Also, this gold standard for *M. gallisepticum* was usually influenced by overgrowth of faster growing *Mycoplasma* species or impeded by other organisms or no growth in subculture. The RT-PCR had been confirmed in a previous study for qualitative and quantitative detection of *M. gallisepticum* (36). The specificity, sensitivity, and reproducibility were sufficient for quantitative detection of *M. gallisepticum* in clinical samples (20).

Neutropenic chickens were used in this study to evade host immunity factors that may play important roles in the *M. gallisepticum* infections as well as the efficacy of antimicrobial therapy. A previous study suggested that the PK/PD index magnitude necessary for successful therapy is reduced in animal models in the presence of neutrophils (38). Therefore, further studies on elucidation of host immunity–pathogen interactions are essential for the drug. Furthermore, a population PK approach should be conducted in the future to derive a population clearance of tiamulin for use in dose calculation. In addition, MIC distribution should be evaluated to take the variation of susceptibility of *M. gallisepticum* to tiamulin into account.

The present study characterized the *in vivo* activity of tiamulin against *M. gallisepticum* in a neutropenic chicken model. The PK/PD surrogate AUC_24h_/MIC correlated well with the *in vivo* antibacterial efficacy. The *in vivo* data suggest that animal dosage regimens should supply AUC_24h_/MIC of tiamulin of 382.68 h for 2 log_10_ ccu equivalents *M. gallisepticum* reduction. These studies suggest that tiamulin, if used for treatment of *M. gallisepticum* infection with an MIC_90_ of 0.03 μg/mL, would benefit from 45 mg/kg i.m. once daily for 3 days.

## Author Contributions

XX, JS, and Y-HL designed this study; TY, XF, JC, and XX carried out the whole experiments; YX helped to analysis the data and revised this manuscript; XX and JS analyzed the data and write this manuscript and Y-HL revised it.

## Conflict of Interest Statement

The authors declare that the research was conducted in the absence of any commercial or financial relationships that could be construed as a potential conflict of interest.
